# Impact of magnetic fields on dose measurement with small ion chambers illustrated in high‐resolution response maps

**DOI:** 10.1002/mp.13591

**Published:** 2019-06-11

**Authors:** Joerg Lehmann, Toby Beveridge, Chris Oliver, Tracy E. Bailey, Jessica E. Lye, Jayde Livingstone, Andrew W. Stevenson, Duncan J. Butler

**Affiliations:** ^1^ Institute of Medical Physics University of Sydney Physics Road A28 Sydney NSW 2006 Australia; ^2^ School of Mathematical and Physical Sciences University of Newcastle Newcastle NSW 2300 Australia; ^3^ Department of Radiation Oncology Calvary Mater Newcastle Newcastle NSW 2300 Australia; ^4^ Australian Radiation Protection and Nuclear Safety Agency (ARPANSA) Yallambie Vic. 3085 Australia; ^5^ Australian Synchrotron 800 Blackburn Road Clayton Vic. 3168 Australia; ^6^ CSIRO Manufacturing Flagship Clayton Vic. 3168 Australia

**Keywords:** ion chamber, magnetic field, MRI linac, response map, Synchrotron

## Abstract

**Purpose:**

Dosimetry of ionizing radiation in the presence of strong magnetic fields is gaining increased relevance in light of advances for MRI‐guided radiation therapy. While the impact of strong magnetic fields on the overall response of ionization chambers has been simulated and measured before, this work investigates the local impact of the magnetic field on dose response in an ion chamber. High‐resolution 1D and 2D response maps have been created for two small clinical thimble ionization chambers, the PinPoint chambers 31006 and 31014 (Physikalisch Technische Werkstaetten Freiburg, Germany).

**Methods:**

Working on the Imaging and Medical Beam Line of the Australian Synchrotron an intense kilovoltage radiation beam with very low divergence, collimated to 0.1 mm was used to scan the chambers by moving them on a 2D motion platform. Measured current and beam position were correlated to create the response maps. Small neodymium magnets were used to create a field of about 0.25 T. Chamber axis, magnetic field, and beam direction were perpendicular to each other. Measurements were performed with both orientations of the magnetic field as well as without it. Chamber biases of 5 and 250 V in both polarities were used.

**Results:**

The local distribution of the response of small thimble‐type ionization chambers was found to be impacted by a magnetic field. Depending on the orientation of the magnetic field, the chamber response near the stem was either enhanced or reduced with the response near the tip behaving the opposite way. Local changes were in the order of up to 40% compared to measurements without the magnetic field present. Bending of the central electrode was observed for the chamber with the steel electrode. The size of the volume of reduced collection near the guard electrode was impacted by the magnetic field.

As the here investigated beam and field parameters differ from those of clinical systems, quantitatively different results would be expected for the latter. However, the gyroradii encountered here were similar to those of a 6–7 MV MRI linac with a 1.5 T magnet.

**Conclusions:**

Magnetic fields impact the performance of ionization chambers also on a local level. For practical measurements this might mean a change in the effective point of measurement, in addition to any global corrections. Further knowledge about the local response will help in selecting or constructing optimized chambers for use in magnetic fields.

## 1 Introduction

Dosimetry of ionizing radiation in the presence of strong magnetic fields has become an area of increased interest with the introduction of radiation therapy treatment systems using Magnetic Resonance Imaging (MRI)‐based image guidance for interfraction and intrafraction patient motion management.^1^, ^2^

The impact of strong magnetic fields, such as the static field of an MRI scanner, on the response of ionization chambers globally has been simulated^3^, ^4^, ^5^ and measured^4^, ^5^, ^6^, ^7^, ^8^. Spindeldreier *et al* 4 have calculated the local spatial response of Farmer‐type chambers in magnetic fields.

The primary goal of this work is to better understand the change in the spatial distribution of charge collection inside an ionization chamber when radiation measurements are made in the presence of a magnetic field. On a practical level, this will hopefully lead to a better approach of measuring radiation dose in MR‐guided radiotherapy treatment systems. Specifically this includes the decision of which ionization chamber (or possibly other detector) to use, as well as guidance on how to design an improved detector in order to optimally assess the dose deposited in the patient. The presented results can also be used to verify Monte Carlo simulations of the same setup, which can then be applied in more complex situations and with different beam energies, where measurements are more difficult or impossible.

## 2 Material and methods

### 2.1 Scanning technique

**Figure 1:**
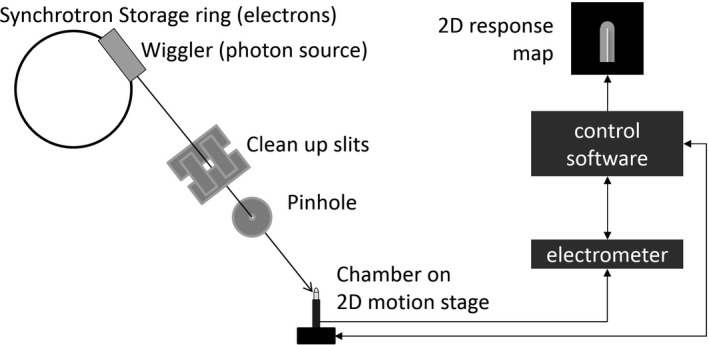
Experimental setup on the Imaging Beam Line of the Australian Synchrotron. Not to scale! The cleanup slits and the pinhole are 0.55 and 0.35 m away from the chamber, respectively. The distance between the photon source (wiggler) and the chamber is 22.35 m. The diameter of the storage ring is 68.76 m.

Work was conducted at the Imaging and Medical Beam Line of the Australian Synchrotron,^9^, ^10^ which produces intense kilovoltage radiation beams with very low divergence. Measurements were performed in Hutch 1B, 22.35 m away from the photon source (Wiggler). A pinhole, which collimated the beam to ~0.1 mm diameter, was placed 35 cm upstream of the measurement location. The beam was scanned across ionization chambers using a technique described earlier.^11^ Figure 1 illustrates the principle components of the measurement setup. Using a high‐precision 2D motion stage the ion chamber was moved with respect to the beam, rather than the other way around, while the current in the chamber was collected with a Keithley 6517A electrometer (Tektronix, USA) and correlated with the position in the control software (Python script). A bias voltage was applied to the chamber as described below.

The main beam parameters are listed in Table 1. The spectrum is also modified to a lesser extent by several thin diamond and beryllium windows, air paths and graphite filters.

**Table 1:** Synchrotron beam parameters

Wiggler magnetic field	3 T
Filter 1 (at ~ 45°)	1 mm Cu
Filter 2 (at ~ 45°)	1 mm Cu
HVL	1.44 mm Cu
Beam energy (average)	95 keV
Pinhole diameter	0.1 mm

The chamber was scanned through the beam in a continuous scan using remote‐controlled stepper motors. The ionization currents were recorded at 60 Hz, to produce a point separation of 0.05 mm. Given the approximate beam diameter of 0.1 mm diameter this was considered sufficient. Collection time ranged from few minutes for 1D scans to 2.5 h for the high‐resolution 2D maps.

### 2.2 Ionization chambers and magnetic field

Two ionization chambers, PTW Pinpoint 31006 and 31014 (Physikalisch Technische Werkstätten Freiburg, Germany) were investigated in this study. They were chosen for their known quality and reliability as well as for their fairly small size. The latter was important as the effort to create a magnetic field increases with the volume for which said field is needed. Additionally, one of the chambers features a steel central electrode while the other one has one made of aluminum, a difference that was thought to be of interest when measuring in a strong magnetic field.

**Figure 2:**
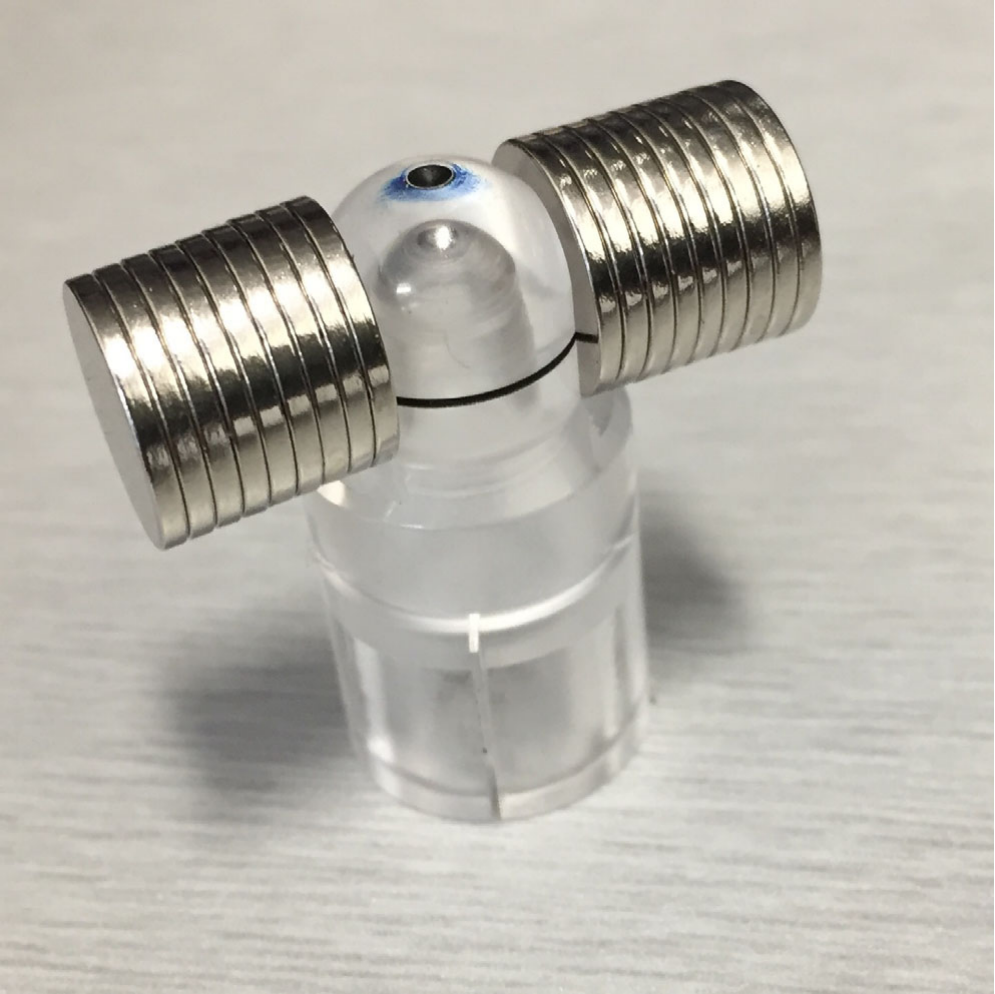
Modified buildup cap for pinpoint ion chamber with neodymium magnets attached. [Color figure can be viewed at http://wileyonlinelibrary.com]

A magnetic field was provided by 18 small neodymium button magnets (10 mm diameter, 1 mm thickness) (OfficeWorks, Australia). The magnets were positioned perpendicular to the chamber axis on either side of the buildup cap (Fig. 2). The cap had been machined flat on either side to accommodate the magnets which held themselves in place due to the magnetic force.

To assess the magnetic field strength experienced by the ion chambers, measurements with a Hall probe (F.W. Bell Gauss/Teslameter Model 5080, OECO LLC, Milwaukie, OR, USA) were performed. Since the probe, which is rectangular in cross‐section, did not fit all the way into the chamber buildup cap used in the experiments, these measurements were performed in a plastic slab setup with space for the probe and the same magnet separation.

In the synchrotron beam chambers were scanned free in air with the modified buildup cap in place and with and without the magnets attached. For some scans the direction of the magnetic field was reversed by switching the magnets.

**Figure 3:**
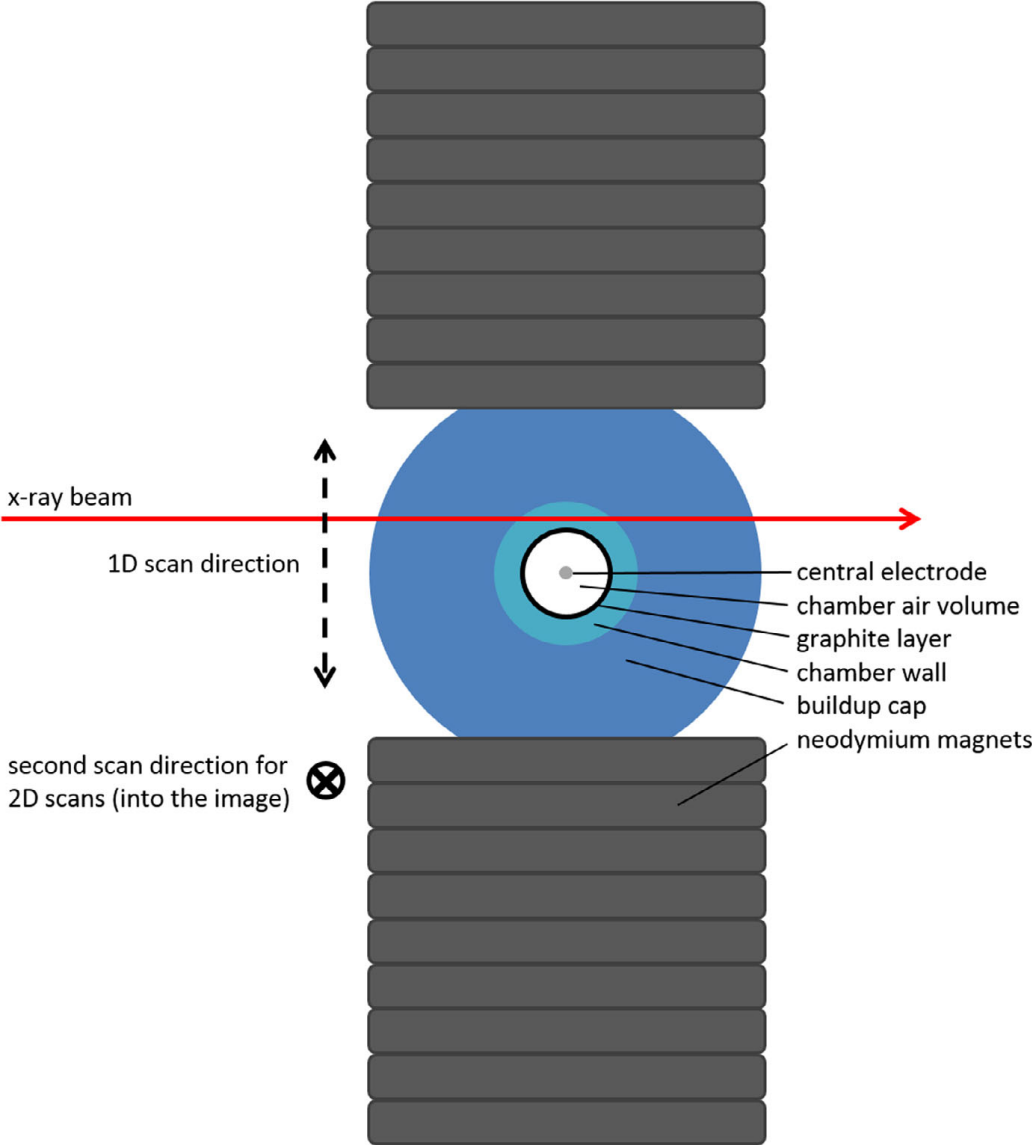
Top view of the scanning setup (cut). [Color figure can be viewed at http://wileyonlinelibrary.com]

As shown in a cut view from above in Fig. 3, the radiation beam (red arrow) was perpendicular to the magnetic field and the chamber axis. Scan direction for 1D profile scans (dashed line double arrow) was parallel to the direction of the magnetic field in the middle of the chamber. These more time‐efficient 1D profiles were used for exploratory measurements and to confirm reproducibility of the results by taking multiple consecutive scans with and without the magnets. 2D response maps were recorded perpendicular to the beam and parallel to the direction of the magnetic field and to axes of the chamber (dashed line double arrow, as above, plus into the image).

The chamber bias voltage was set to the default 250 V as well as to 5 V. The latter was chosen to explore an enhanced effect. Measurements were performed with both polarities.

Measurements with nonmagnetic metal in place of the magnets have been performed for a similar measurement setup to confirm that the presence of metal did not impact the pattern of charge deposition in the ionization chamber.

## 3 Results

Measurements of the magnetic field strength indicated a field of about 0.25 T in the air volume of the ionization chamber. While the magnets with a 10‐mm diameter measured twice the length of the air cavity of the chambers, there was still some gradual falloff of the magnetic field toward the ends of the chamber with the field strength at the very end of the chamber decreasing down to 88% of that at its center.

Line scans of current vs position along the midline of a thimble ion chamber perpendicular to its main axis feature small increases in current on the inside of the chamber walls and a larger double peak at the electrode. This has been observed and described previously^11^ and will be assumed to be understood here. The same applies to the stronger response around the electrode for steel vs aluminum. This work focuses on relative changes to this response caused by the presence of a magnetic field.

**Figure 4:**
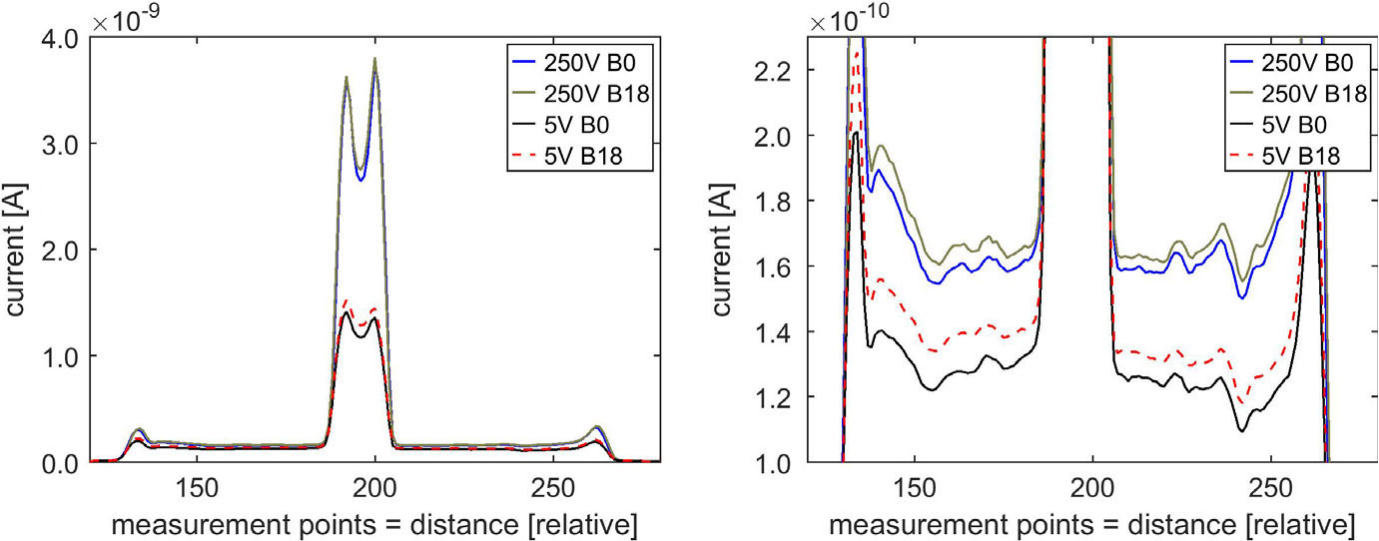
1D scans along the midline of a PTW31006 PinPoint chamber (steel central electrode). 5 and 250 V bias, without (B0) and with magnetic field (B18 = 18 magnets). Each plot is an average of three scans. Right image is a zoomed in view. [Color figure can be viewed at http://wileyonlinelibrary.com]

Using a standard bias voltage of 250 V 1D scans along the midline of the chamber with the steel electrode showed a small difference between scans taken with and without the magnetic field applied. This effect was found to be enhanced in the measurements with the lower bias voltage of 5 V (Fig. 4). Interestingly the current readings outside central electrode and chamber wall are distinctly correlated for the four plots (Fig. 4, right), each of which represents the average of three measurements. Due to time restrictions, 2D scans were first performed for 5 V cases and then for selected 250 V cases.

**Figure 5:**
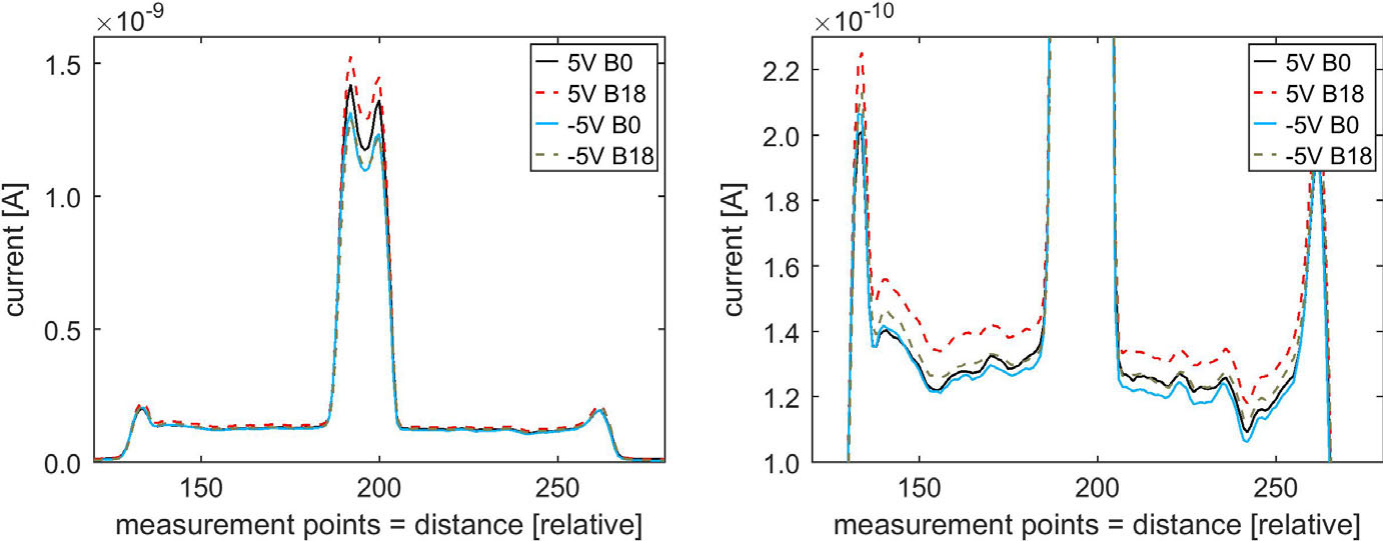
1D scans along the midline of a PTW31006 PinPoint chamber (steel central electrode). 5 and −5 V bias, without (B0) and with magnetic field (B18). Each plot is an average of three scans. Right image is a zoomed in view. [Color figure can be viewed at http://wileyonlinelibrary.com]

Investigating the impact of the polarity of the bias showed a stronger effect for +5 V compared to −5 V (Fig. 5).

**Figure 6:**
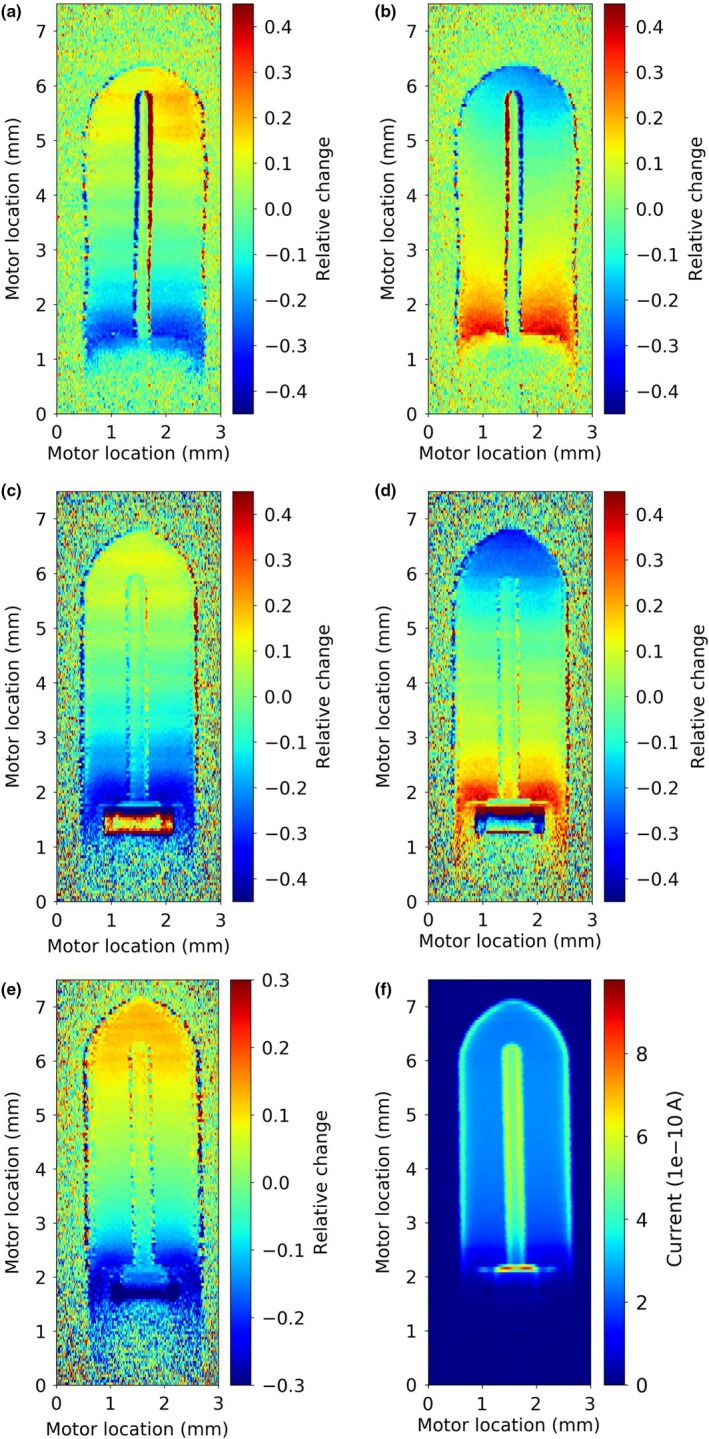
2D response maps of a PTW31006 PinPoint chamber featuring a steel central electrode (plates a and b), and a PTW31014 PinPoint chamber with an aluminum central electrode (plates c–f), displayed as relative current for measurements with and without a 0.25 T magnetic field applied (plates a–e) and absolute current with the 0.25 T magnetic field applied (plate f). Direction of the magnetic field is from left to right of each image (or vice versa), direction of the radiation beam is into the image. Chamber bias was 5 V (plates a–d), and 250 V (plates e–f). [Color figure can be viewed at http://wileyonlinelibrary.com]

Using the 5 V bias setting, 2D scans obtained with and without the magnetic field showed differences larger than 40% toward the ends of the steel electrode chamber. Little change was seen in the middle part of the chamber, where the 1D scans had been taken. Reversing the magnetic field produced an effect of the same quality in the opposite direction. [Fig. 6(a) and 6(b)].

For the chamber with the aluminum electrode the 2D response maps, again with 5 V bias, showed a comparably weaker signal around the electrode, as previously reported and not shown here, but the same relative signal change inside the chamber air volume when the magnetic field was applied as found in the chamber with the steel electrode. [Fig. 6(c) and 6(d)] Larger differences can be seen around the electrode holder.

For the generally used bias of 250 V, the relative changes seen in the 2D scan are similar to those observed with the 5 V bias but of a lower magnitude, up to about 25%. This is shown for the chamber with the aluminum electrode (PTW 31014) in Fig. 6(e). Figure 6(f) shows the actual current measured in that chamber with 250 V bias.

## 4 Discussion

The local distribution of the response of small thimble‐type ionization chambers was found to be impacted by a magnetic field for the commonly investigated geometry where chamber axis, magnetic field, and beam direction are perpendicular to each other. Depending on the orientation of the magnetic field the chamber response near the stem, here represented as measured current, was either enhanced or reduced with the response near the tip behaving the opposite way.

**Figure 7:**
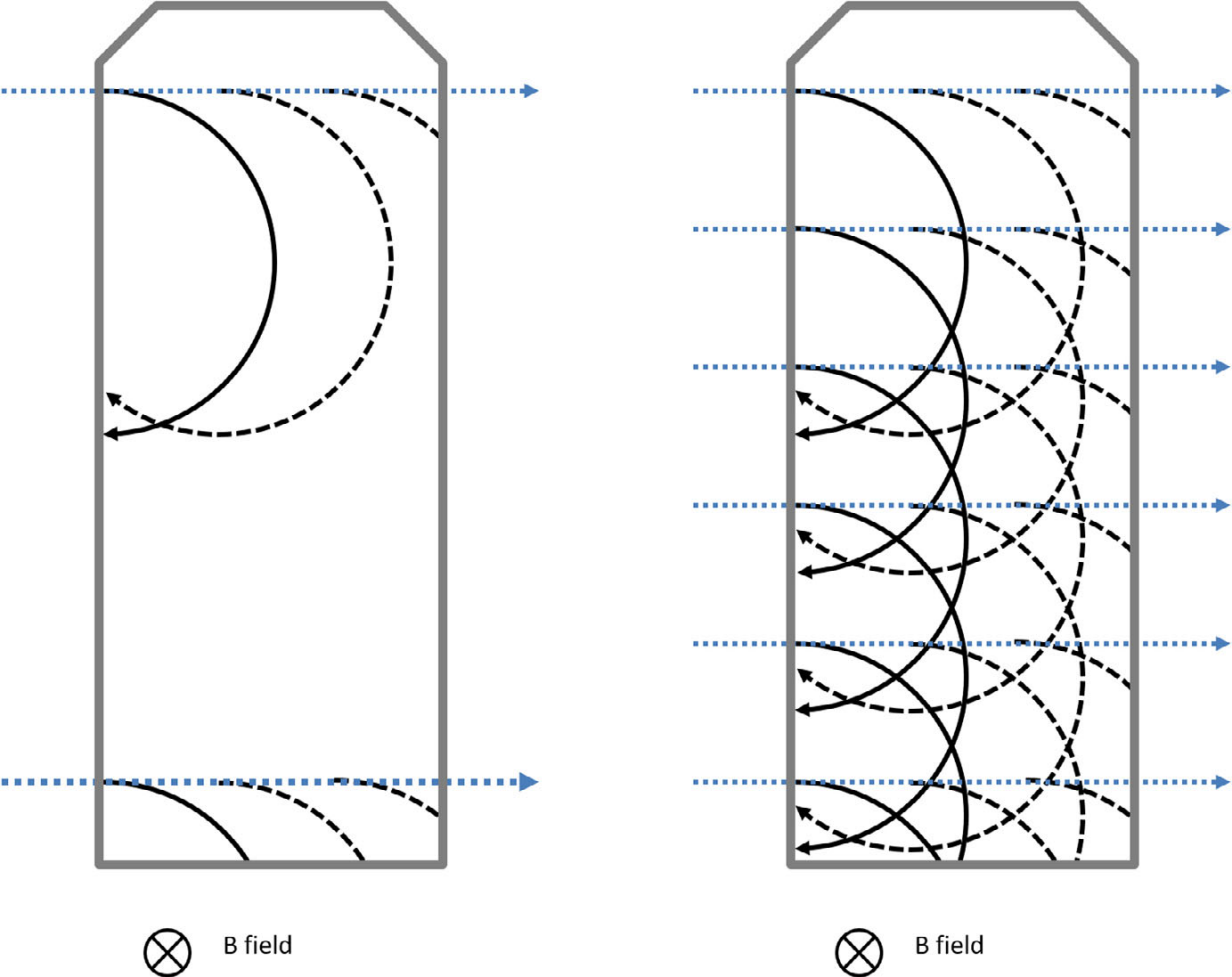
Simplified schematic of the impact of the magnetic field on the secondary electrons when photons (dotted lines) pass through an ionization chamber, shown for single photons on top and bottom of the chamber (left) and a number of photons distributed over the length of the chamber (right). Like reported by others^5^ electrons set in motion in the phantom or chamber wall will be impacted in their path by the magnetic field (solid line). In case of the here used kV energy range, additional electrons will be set free while the photon travels through the air cavity. These electrons (dashed lines) will be impacted similarly. [Color figure can be viewed at http://wileyonlinelibrary.com]

**Figure 8:**
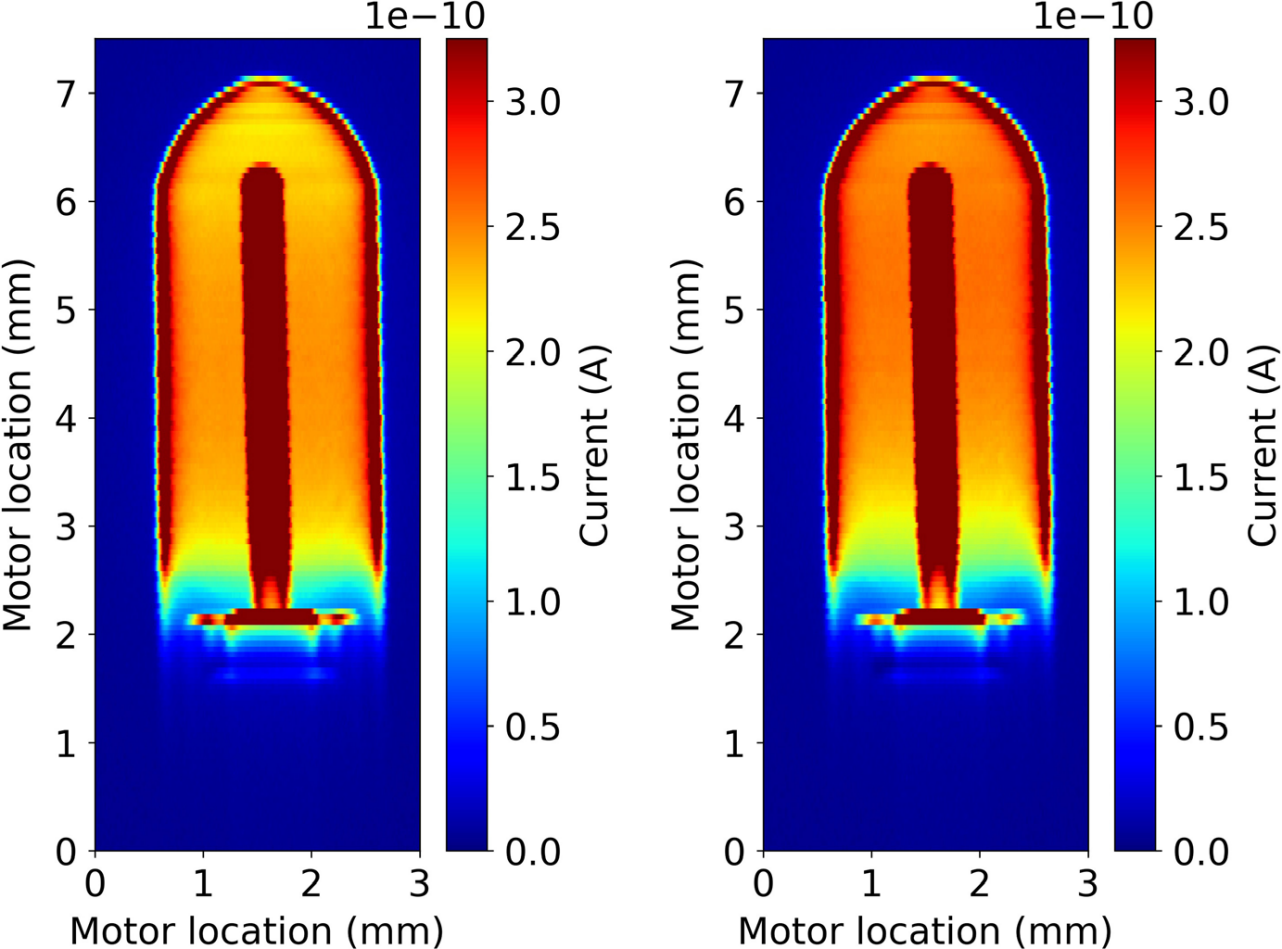
Saturated 2D response maps of a PTW31014 PinPoint chamber featuring an aluminum central electrode displayed as absolute current for measurements without (left) and with a 0.25 T magnetic field applied (right). Chamber bias was 250 V. Note that the employed saturation, which enables appreciation of the differences within the air volume renders the display in the saturated dark red areas not meaningful. [Color figure can be viewed at http://wileyonlinelibrary.com]

These distributions can be explained by the change in pattern of the movement of the electrons in the presence of the magnetic field compared with the generally forward and equally lateral movement in the absence of such field: The paths of the secondary electrons are bent by the magnetic field as described by Meijsing et al, shown in Fig. 8 of their work 5 and illustrated with the solid lines in Fig. 7 here. The radii of the curvature of the path of the electrons, also referred to as gyro radii, which increase with the energy of the electrons and decrease with the magnetic field strength, were found to be in the range of a few millimeters. This is similar to those at an MR‐linac, where both energy and magnetic field strength are higher (1.5 T, 6–7 MV) (Table 2). As the radii are similar to the dimension of the ionization chamber they would be expected to impact performance. In the here presented measurements, because of the photon energy however, many more additional electrons are set in motion while a photon passes through the chamber. These electrons will also be affected in their paths by the magnetic field as indicated with the dashed lines in Fig. 7. When the small beam in these measurements hit the top part of the chamber the electrons will travel on a longer path because of the magnetic field in the shown arrangement compared to the situation without the magnetic field (Fig. 7, left). The longer path leads to more ionizations and a higher signal (current) measured. When the beam hits the bottom part of the chamber, the electrons are directed outside, travel a shorter distance, and hence create a lower signal. This matches the observations in Fig. 6(a), 6(c), and 6(e). Overall the shown field orientation leads to more electron path lengths in the lower part of the chamber. (Fig. 7, right). Therefore a traditional Monte Carlo simulation that reports deposited dose in various regions of the chamber would report a higher dose in the lower part of the chamber, opposite to what was shown here. Neither approach is more or less correct, each just reports different quantities.

**Table 2:** Calculated radii of curvature for electron paths in the presence of magnetic fields

Electron energy [MeV]	Radius of curvature [mm] for selected magnetic field strengths					
	0.25 T	0.35 T	0.75 T	0.9 T	1 T	1.5 T
0.01	1.3	1.0	0.4	0.4	0.3	0.2
0.05	3.0	2.2	1.0	0.8	0.8	0.5
0.1	4.3	3.0	1.4	1.2	1.1	0.7
1	13.5	9.6	4.5	3.7	3.4	2.2
2	19.1	13.6	6.4	5.3	4.8	3.2
6	33.0	23.6	11.0	9.2	8.3	5.5

The distributions in Fig. 6(b) and 6(d) can be explained with the same principles and a reversed magnetic field.

The found effects correspond with findings from the Monte Carlo simulation by Malkov and Rogers,^12^ although their work focused on the stem side of the chamber. As described by them, the effect is energy dependent and the here investigated scenario of a ~100 keV beam likely produces larger disturbances than clinical beams in the MV range will.

The results are also in broad agreement with the effects predicted by Spindeldreier *et al.,* 4 although differences in the energy, field, and chamber geometry preclude a quantitative comparison. However, as described above regarding Monte Carlo simulations, it needs to be noted that Spindeldreier et al report 2D dose maps of ionization chambers in magnetic fields for a large photon field and the coordinates in the maps mark the position of the azimuthal rings around the chamber axis where the dose was deposited inside the sensitive volume. Additionally, dose values were scored irrespective of actual collection via the electrodes and circuitry. In the further analysis, this was qualified by excluding some “dead volumes”. In the here presented results, the map is a 2D projection of actually collected charge and the coordinates in the 2D maps mark the entrance of the 0.1 mm beam, stepped in 0.05 mm steps. Given the low dispersion of the beam a conceptual comparison is considered valid. The presence of “dead volumes,” which was also discussed by Looe et al.^13^, ^14^, ^15^ for a different chamber, has been confirmed [Fig. 6(f)] and can be better appreciated in Fig. 8, where the display has been saturated in the high current areas for improved differentiation of the currents collected in the air volume. It appears that there is no strict boundary between functioning collecting volume and “dead” volume, but a gradual change over almost 1 mm in the direction of the chamber axis, which changes slightly in the presence of the magnetic field.

The magnetic field from the permanent magnets used in this study shows a small gradual falloff towards the edges of the chamber, which would not be the case with the field of a commercial MRI device. Given the location of the observed effects this does not take away from the validity of the results. Additionally, different susceptibility of the materials of the components of the chamber would likely have added some small local disturbances of the magnetic field. While the majority of the material is PMMA, which is very MR compatible and would not disturb the field,^16^ the ferro metallic parts, in particular the steel electrode of the 31006 chamber would have some impact on the field. There was little difference observed between the relative chamber responses inside the air volume of the two chambers which had different central electrode materials and also a slightly different thimble shapes. This indicates that any changes in the magnetic field due to the different magnetic susceptibility of aluminum and steel have only a minor and very local effect. Overall, any local disturbance of the field would be similar to what would happens in a real measurement scenario at a MR‐guided treatment system and hence in line with the goal of this study.

Lower bias voltages were found to increase the local difference between measurement without and with the magnetic field present. This was seen for the 1D investigations (Fig. 4) as well as for the 2D plots (Fig. 6). As in practical applications bias voltages are generally chosen as high as possible for highest collection efficiency, this might only be of limited use. However, if the dependence of chamber performance as a function of the bias voltage differs with and without a magnetic field present, this could potentially be exploited to correct for the effect of the magnetic field. More work will be needed in this area. Likewise, changing the polarity of the bias resulted in some markedly different responses in 1D investigations (Fig. 5), without fully revealing the underlying mechanism. The above discussed volumes of low or no collection (“dead volumes” — 4, 14) might not be the final or complete answer and the bias voltage will likely be a valuable tool in the search for it.

The steel electrode of the PTW31006 PinPoint chamber appeared to bend slightly under the influence of the magnetic field, as judged by the recorded response. While conceptually not unexpected, this finding is interesting in that it could mean a change in the effective point of measurement of the chamber, especially with stronger fields. A bending electrode would also need special attention in a Monte Carlo simulation.

There is some more structure in stem region of the difference plot for the 31014 chamber (aluminium central electrode) than the 31006 chamber (steel central electrode). Because the B field affects signal arising in the stem, it is possible that the global response of the chamber at opposite bias voltages may also be B field dependent.

## 5 Conclusions

Magnetic fields impact the response of ionization chambers on a local level. The knowledge of where the response changes should be helpful in understanding the response of the whole chamber, and might have other implications, for example, this might mean a change in the effective point of measurement, in addition to any global corrections. Further knowledge about the local response will help selecting or constructing optimized chambers for use in magnetic fields.

## Conflicts of interest

The authors have no relevant conflicts of interest to disclose.
